# Effect of preoperative neuromuscular training (NEMEX-TJR) on functional outcome after total knee replacement: an assessor-blinded randomized controlled trial

**DOI:** 10.1186/s12891-015-0556-8

**Published:** 2015-04-25

**Authors:** Erika O Huber, Ewa M Roos, André Meichtry, Rob A de Bie, Heike A Bischoff-Ferrari

**Affiliations:** Centre of Aging and Mobility, University Hospital Zurich and Waid City Hospital Zurich, Rämistrasse 100, Zurich, Switzerland; School of Health Professions, Institute of Physiotherapy, Zurich University of Applied Sciences, Technikumstrasse 71, 8401 Winterthur, Switzerland; Department of Epidemiology, Musculoskeletal Research Division CAHPRI, Maastricht University, PO Box 616, Maastricht, The Netherlands; University of Southern Denmark, Institute of Sports Science and Clinical Biomechanics, Campusvej 55, 5230 Odense M, Denmark; Department of Geriatrics and Aging Research, University Hospital Zurich, Rämistrasse 100, Zurich, Switzerland

**Keywords:** Neuromuscular training, Preoperative, Knee osteoarthritis, Total knee replacement, Randomized controlled trial

## Abstract

**Background:**

Improving functional status preoperatively through exercise may improve postoperative outcome. Previous knowledge on preoperative exercise in knee osteoarthritis is insufficient. The aim of the study was to compare the difference in change between groups in lower extremity function from baseline to 3 months after Total Knee Replacement (TKR) following a neuromuscular exercise programme (NEMEX-TJR) plus a knee school educational package (KS) or KS alone.

**Methods:**

45 patients (55–83 years, 53% male, waiting for TKR) were randomized to receive a minimum of 8 sessions of NEMEXTJR plus 3 sessions of KS or 3 sessions of KS alone. Function was assessed with the Chair Stand Test (CST, primary endpoint) and the Knee Injury and Osteoarthritis Outcome Score (KOOS) subscales focusing on daily living function (ADL) and pain (secondary endpoints). Assessments were performed immediately before and after the intervention, and at 6 weeks, 3 months and 12 months after surgery by a physiotherapist, blinded to group allocation.

**Results:**

After intervention before surgery we observed a small improvement for primary and secondary endpoints in both groups, which did not differ significantly between groups: comparing the exercise to the control group the treatment effect for the CST was −1.5 seconds (95% CI: −5.3, 2.2), for KOOS ADL and KOOS pain the treatment effect was 1.3 points (−10.1, 12.8) and −2.3 (−12.4, 7.9) respectively. At 3 months after surgery we observed a small improvement in the primary endpoint in the control group and a significant improvement in the secondary endpoints in both exercise and control groups, which did not differ significantly between groups: comparing the exercise group to the control group the treatment effect in the CST was 2.0 seconds (−1.8, 5.8), for KOOS ADL and KOOS pain the treatment effect was −4.9 points (−16.3, 6.5) and −3.3 points (−13.5, 6.8) respectively.

**Conclusions:**

A median (IQR) of 10 (8, 14) exercise sessions before surgery showed an additional small but non-significant improvement in all functional assessments compared to patient education alone. These benefits were not sustained after TKR. Our trial doesn’t give a conclusive answer to whether additional preoperative exercise on postoperative functional outcomes is beneficial.

**Electronic supplementary material:**

The online version of this article (doi:10.1186/s12891-015-0556-8) contains supplementary material, which is available to authorized users.

## Background

Osteoarthritis (OA) is the second most common diagnosis made in older adults seeking medical care [1] and the leading cause of disability at older age [2]. For sufferers from severe knee OA, Total Knee Replacement (TKR) is the preferred treatment option to significantly improve function and pain [3,4]. Given the growing segment of the senior population in the Western World, the rate of these procedures will rise exponentially over the next decade. This will result in high health-care expenditures, due to an absolute increase in TKR surgery [5].

Most patients undergoing TKR experience pain-relief [6,7], but up to 30% of patients continue to have significant pain and functional problems after TKR [8-12]. These continuing problems might be addressed through, (a) fostering postoperative functional recovery by offering a preoperative exercise program to optimise the preoperative functional status of patients awaiting TKR [13] and (b) modifying patients’ expectations by offering an educational program before surgery [14].

The most recent review and meta-analysis on preoperative exercise on functional recovery after joint replacement was published in 2012 and included 12 trials [15]. The authors concluded that “preoperative therapeutic exercise for total joint replacement did not demonstrate beneficial effects on postoperative functional recovery. However, poor therapeutic validity of the therapeutic exercise programmes may have hampered potentially beneficial effects”.

Exercise programs should include mixed activities, including aerobic, strength and proprioceptive exercises [16]. Sensorimotor deficiencies, in terms of lower limb muscle weakness and altered muscle activation patterns, should be addressed specifically [17]. A well-described exercise programme that addresses these deficiencies, and previously found to be feasible in OA patients waitlisted for TKR, is the neuromuscular exercise programme-total joint replacement (NEMEX-TJR) [18].

Alternatively, patients may have to be better informed and take more responsibility for their care and need to be prepared for both the surgical procedure and the recovery period in advance [19]. An educational program during the waiting period for TKR may help patients to prepare themselves for their rehabilitation after surgery [20] and modify patients’ preoperative expectations [14].

### Objectives

To study the effect of a preoperative neuromuscular training (NEMEX-TJR) plus knee school educational program (KS) compared to the KS alone on lower extremity function and pain in individuals aged 55–90 years on a waiting list for TKR due to severe knee OA.

### Hypothesis

Primary endpoint: we hypothesize that patients undergoing the NEMEX-TJR in addition to the KS will be quicker in performing the Chair Stand Test (performance test of lower extremity function) compared with those receiving the KS alone, both when measured immediately after the intervention and 3 months after TKR surgery.

Secondary endpoints: we hypothesize that patients undergoing the NEMEX-TJR in addition to the KS will have a greater improvement in the patient-reported outcome (PRO) measure KOOS (subscales ADL function and pain) compared with those receiving the KS alone, when measured immediately after the intervention as well as 6 weeks, 3 months and 1 year after TKR surgery.

## Methods

### Study design

The study design was an assessor-blinded randomized controlled trial. Outcomes were measured at baseline (6–12 weeks preoperative), 1 week preoperative, 6 weeks postoperative, 3 months postoperative and 12 months postoperative, with the primary endpoint being 3 months postoperative. Ethical approval was granted by the Ethics Committee of the Cantons Aargau and Solothurn, Switzerland, approval number 2009/12 and the trial is registered with ClinicalTrials.gov, identifier: NCT00913575.

### Participants and randomization

Participants were eligible when: they were on a waiting list for primary TKR at the Cantonal Hospital Olten or the Cantonal Hospital Aarau and sufficient time existed before the operation date in order to take at minimum 8 sessions of the training program; aged 55 to 90 years; understood German; and lived at home. Originally it was planned to only include patients aged 60 and above. However, during the recruitment phase the age was lowered to 55 to increase the number of participants. The Ethics Committee accepted an amendment under the same approval number. Exclusion criteria were: revision surgery; history of inflammatory arthritis; cognitive impairments; absence before or after surgery; and inability to walk at least 3 meters with or without a walking aid. Although our last exclusion criteria defined a low threshold for functionality to be enrolled in the trial, none of the study participants was unable to perform the lower extremity tests defined in the protocol. Recruitment and eligibility assessment were conducted by the orthopaedic surgeon at the time the patient was placed on the waiting list. Eligible patients were referred to the study centre (Centre on Aging and Mobility, University of Zurich) by fax. After confirming their interest, eligible individuals received detailed participant information about the study procedure. Informed written consent was obtained on the day of baseline assessment.

Allocation was concealed and conducted by a study nurse of the independent randomization centre after baseline assessment. Participants were randomized using block allocation with a block size of four from a computer generated list. Allocation to the intervention or control group was performed by telephone.

### Surgical and rehabilitation procedure

All operations were performed by four senior orthopaedic surgeons, using a standard anterior skin incision followed by medial parapatellar arthrotomy, in an effort to minimize disruption in the medial lymphatic and saphenous nerve branches. The implants used were all a posterior cruciate ligament-retaining system performed in a femur first technique with measured femoral sizing and rotation. The implants were fully cemented using a second generation sandwich technique.

The post-surgical rehabilitation process followed an individualised treatment plan. In the acute care hospital, physiotherapy treatments aimed to improve passive and active range of motion, to reduce swelling and to improve walking capacity with canes. Patients were discharged after 7–10 days. The post-acute rehabilitation took place either in an outpatient physiotherapy practice (ranging from 9–18 treatment sessions) or in an inpatient rehabilitation clinic (on average 2–3 weeks, corresponding to 10–15 treatment days with at least two treatment sessions per day). At both sites treatments aimed to improve active range of motion, muscle strength and activities of daily living, such as walking capacity and climbing stairs”.

### Interventions

Patients of the intervention group attended knee school preoperatively (starting about 4 weeks before surgery) and a neuromuscular training program (for 4–12 weeks, depending on their location on the waiting list for surgery). Patients of the control group attended only the three sessions of knee school.

The neuromuscular training followed the principles of neuromuscular and biomechanical training as described in the neuromuscular training method (neuromuscular exercise programme-total joint replacement, NEMEX-TJR) [18]. The programme is feasible in patients with severe hip or knee OA, in terms of safe self-reported pain following training, decreased or unchanged pain during the training period, few joint-specific adverse events, and achieved progression of training level during the training period. In a controlled before-and-after study NEMEX-TJR (mean 12 weeks (SD 5.6) of training) improved self-reported outcomes (7-20%) and physical function (5-19%) (p < 0.005) [21]. Between 39% and 61% of knee patients displayed a clinically meaningful improvement (≥15%) in KOOS subscales through the training.

The training took place in groups under the supervision of an experienced, specially-trained physiotherapist and consisted of a 10-minute aerobic warm-up on a stationary exercise bike, followed by a four-exercise circuit programme, and finishing with a cool down period of about 10 minutes.

The key elements of the circuit programme were stability/postural function, functional alignment, lower-extremity muscle strength and functional exercises. Each exercise was performed for 10–15 repetitions and for 2–3 cycles, with rest between each exercise and cycle. To allow progression, three levels of difficulty were defined. Progression was provided by varying the number, direction and velocity of the movements, by increasing the load and/or by changing the support surface. Progression was made when an exercise could be performed with 15 repetitions and 3 cycles with good neuromuscular control and good quality of performance (based on visual inspection by the physiotherapist) and with minimal exertion and control of the movement (perceived by the patient). Training programme documentation included the number of training sessions, level of difficulty per session, pain on a 0 to 10 scale before and after each session and 24 hours after each session.

The knee school was taught by an experienced and specially-trained physiotherapist over 3 individual or group sessions, one session per week, starting about 4 weeks before the operation. Knee school sessions were separately organised for participants of the intervention group and those of the control group to avoid contamination. The content of the knee school included information on anatomy of the knee joint and adjacent functional structures, recommended activities with prosthesis and post-operative pain management, and details on the post-operative rehabilitation phase. Didactical elements included models of the knee joint and the lower extremity, working sheets, photos and videos, handouts, PowerPoint presentations and peer discussions.

A description of the intervention has been published previously, where the details of the program are described in the additional file [22].

### Assessment

Outcomes were measured at baseline (6–12 weeks preoperative, all measures), 1 week preoperative (after the intervention, all measures), 6 weeks postoperative (PROs only), 3 months postoperative (all measures) and 1 year postoperative (PROs only). A special clinical examination room with standardized equipment was used to perform all the measures at all time-points at both sites. Two experienced physiotherapists (assessors), not working at the recruitment sites and not involved in the neuromuscular training and the knee school, had been specifically trained for the assessments in this study and were blinded to group allocation. Participants were instructed not to mention the allocation.

### Outcomes

The effect of the intervention on lower extremity function was evaluated by performance tests and by PRO measures. In the trial registration we chose as primary endpoints the Chair Stand Test and the KOOS subscales ADL and pain. During the recruitment phase we made adaptions in our endpoints for clearer interpretability, keeping only the Chair Stand Test as primary endpoint. The KOOS subscales became secondary endpoint and all other measures became additional outcome measures.

#### Primary outcome measure

The primary outcome is the Chair Stand Test, also known as the repeated sit-to-stand test. It is commonly used as a measure of lower extremity strength, balance and reaction time [23-25]. The time required for five repetitions of rising from a chair and sitting down again was performed according to the OsteoArthritis Initiative manual, including detailed standardization and instructions (available from: http://oai.epi-ucsf.org). Patients sat on a standard chair without armrests. Feet were placed comfortably on the floor with knees flexed slightly greater than 90 degrees. Patients were asked to stand up to a fully erect standing position five times as quickly as possible without using their hands (arms folded across the chest). Timing with a stopwatch started on “Go” (after a countdown from 3) and ended on the fifth stand. After an exercise phase, the test was performed once. The Chair Stand Test is easy to perform in clinical practice and has shown excellent intra- and inter-rater reliability (ICC, 0.89) in patients with severe hip or knee OA [26]. The Chair Stand Test was also found to predict disability across populations accurately [27].

#### Secondary outcome measure

Secondary outcomes are knee pain and function, assessed by the KOOS questionnaire. The KOOS is a commonly used patient-reported outcome with overall acceptable psychometric properties to evaluate patients with knee injury and knee OA [28], including those having TKR [29]. KOOS contains 5 subscales with a total of 42 items: 1) pain; 2) other symptoms; 3) function in daily living (ADL); 4) function in sport and recreation (Sport/Rec); and 5) knee-related quality of life (QOL). Each question receives a score from 0 to 4 and the scores are transformed to a 0 to 100 score (0 = extreme symptoms, 100 = no symptoms). Since exercise training is aiming to improve function, we are particularly interested in the KOOS ADL subscale for the functional outcome measure.

The German version of the KOOS was used in this trial [30]. The User’s Guide, including scoring instructions, are available from http://www.koos.nu.

#### Additional outcome measures

Lower limb function:

KOOS subscales other symptoms, Sport/Rec and QOL, assessed by the KOOS questionnaire [30]. Isometric muscle strength of knee flexors and extensors, measured with a hand-held pull gauge [31,32].The ability to alternate rapidly between concentric and eccentric work of the extensor muscles of the hip and knee is impaired in many patients with knee OA [33]. The ability of rapid alternation between concentric and eccentric function is measured using maximal number of knee-bending in 30 seconds, which is a valid and reliable test (ICC, 0.96) [34]. Range of Motion is measured with a long-arm goniometer [35]. Walking speed is assessed with the 20 m walk test (ICC, 0.93) [26], a reliable modification of the short walk test used in many epidemiological and clinical studies. The test measures the time it takes to walk 20 meters at the participant’s usual walking pace, along with the number of steps that they take to walk 20 meters [36]. Lower extremity mobility is further assessed with the Timed Up and Go test, which requires a person to rise from a standard stair, walk to a line that is 3 meters away, turn 180 degrees, return to the chair and sit down [37].

Physical activity and health-related quality of life:

Physical activity is measured by the SenseWear armband, a device for quantifying physical activity in daily life [38,39]. It collects the following data: energy expenditure, average MET’s, physical activity duration, steps per day and the physical activity distribution (sedentary, moderate, vigorous and very vigorous). In addition, physical activity is measured by 10 activity questions in NHANES III [40], from which MET values can be calculated [41]. Health-related quality of life is measured by the generic questionnaire SF-36 [42,43]. General health status is measured by the EuroQoL (EQ-5D, including EQ-VAS). The EQ-5D is used to complement the SF-36, allowing health economic evaluation and comparison to other knee OA populations [44].

### Sample size calculation

The sample size calculation is based on the primary endpoint - the Chair Stand Test. We assume that the mean difference in change over time between groups is 7.3 seconds (corresponding to means of 8.3 and 1.0, respectively) and the common within-group standard deviation is 7.3. This effect was selected based on pilot data of an uncontrolled trial in knee OA patients, assuming that our control group (without exercise training) would not improve over time while awaiting TKR. It is also assumed that the effect size is reasonable, in the sense that an effect of this magnitude could be anticipated in this field of research. Alpha value has been set at 0.05 and the power has been set at 0.9. In each group 25 patients are needed. Assuming a drop-out rate of 12%, we will include 40 patients per group.

### Statistical analysis

The data for primary, secondary and additional outcomes were analysed according to the intention-to-treat principle, including all randomized individuals. Descriptive statistics were evaluated for patient characteristics. For baseline between-group comparisons, we performed the Wilcoxon-RankSum Test for continuous and the Chi squared test for categorical variables.

For each outcome, we fitted a linear mixed model (LMM) to the data with time, group and group-time interaction as fixed effects and subject as a random intercept. In a primary analysis we adjusted for gender, BMI and age. Random intercept models are equivalent to repeated measures ANOVA and take into account the correlation between repeated measurements. In contrast to classical repeated measures ANOVA, they can deal better with missing observations while retaining power. For the details of the model see Additional file 1.

Our primary interest was in the treatment effects, which are equivalent to the group-time interactions. Likelihood ratio tests for nested models were performed for model selection. We controlled for the potential confounding factors and tested whether age, gender or BMI could be removed from the model. Specific contrasts such as within-group changes and group-time interactions between specific time points were estimated. All simultaneous inference procedures controlled the family-wise error rate of α = 0.05. Residual analysis was performed to check model assumptions.

All analyses were conducted with R version 2.14.1 software [45,46].

## Results

From May 2009 to June 2012 a total of 72 patients were eligible, of those 27 declined participation and 45 patients were assessed and randomized to the two groups. The 22 patients in the exercise group and the 23 patients in the control group were comparable with respect to age, gender, BMI, time to surgery and baseline assessments without significant differences in any of the baseline characteristics outlined in Table 1.

**Table 1:** **Baseline characteristics of study participants**

	**Intervention group (n = 22)**	**Control group (n = 23)**	***p*** **-value**
Age – years	68.8 ± 8.0	71.9 ± 8.1	0.198
Females (%)	11 (50)	10 (43.5)	0.889
BMI – kg/m^2^	30.8 ± 4.9	29.9 ± 5.5	0.507
Time to surgery - weeks	8.9 ± 3.6	8.5 ± 3.0	0.790
**Primary and secondary outcome measures**			
Chair Stand Test	16.3 ± 8.3	16.0 ± 4.7	0.581
KOOS pain	48.1 ± 17.6	47.3 ± 16.8	0.864
KOOS ADL	51.7 ± 17.8	49.9 ± 19.1	0.716
**Additional outcome measures**			
KOOS symptoms	47.4 ± 13.5	49.1 ± 15.1	0.982
KOOS sport and recreation	18.3 ± 17.5	16.9 ± 17.6	0.680
KOOS quality of life	26.4 ± 14.5	26.8 ± 15.3	0.900
Muscle strength			
Knee extension op	244.8 ± 91.7	247.6 ± 100.5	1.000
Knee extension contra	285.0 ± 97.8	315.4 ± 123.1	0.386
Knee flexion op	165.4 ± 61.9	151.9 ± 53.8	0.447
Knee flexion contra	176.6 ± 65.6	185.4 ± 66.9	0.525
Knee-bendings/30s	13.1 ± 3.9	11.4 ± 5.5	0.406
ROM of the knee			
Flexion op	115.7 ± 11.5	115.0 ± 12.0	0.748
Flexion contra	119.5 ± 8.0	122.9 ± 11.5	0.219
Extension op	6.6 ± 4.7	6.7 ± 4.4	0.708
Extension contra	1.6 ± 2.8	3.0 ± 3.9	0.203
20 m walk test	17.6 ± 2.9	17.9 ± 4.8	0.658
Timed up and go	9.7 ± 2.4	11.5 ± 7.2	0.329
Physical activity level			
METs (kcal/h/kg)	28.5 ± 4.5	29.3 ± 4.5	0.617
Steps (daily average)	6158.7 ± 3317.4	6145.0 ± 2810.9	0.967
Adapted NHANES III METs	24.4 ± 23.6	21.2 ± 29.0	0.244
SF 36			
Physical functioning	43.4 ± 17.5	44.1 ± 19.1	0.906
Role physical	43.2 ± 45.8	35.2 ± 44.8	0.575
Bodily pain	41.9 ± 15.5	43.7 ± 14.3	0.706
General health	62.6 ± 15.2	64.4 ± 17.4	0.467
Vitality	57.1 ± 20.3	52.3 ± 19.4	0.723
Social functioning	87.5 ± 18.1	83.5 ± 22.6	0.580
Role emotional	77.3 ± 40.4	62.1 ± 47.5	0.305
Mental health	79.5 ± 14.2	73.6 ± 16.6	0.215
EQ-5D			
Mobility	1.7 ± 0.5	1.7 ± 0.5	0.755
Self-care	1.0 ± 0.0	1.1 ± 0.4	0.081
Usual Activities	1.4 ± 0.5	1.3 ± 0.5	0.764
Pain/discomfort	2.1 ± 0.4	2.0 ± 0.2	0.335
Anxiety/Depression	1.2 ± 0.4	1.2 ± 0.4	0.724
EQ-VAS	64.6 ± 18.2	67.7 ± 16.0	0.684
RAPT	9.4 ± 1.5	9.4 ± 1.4	0.298

Values are the mean ± SD for continuous variables (Wilcoxon Rank Sum test) and the number (percentage) for categorical variables (Chi-square test). There were no significant between group differences for all variables.

Legend:

BMI, body mass index; op, operated leg; contra, contralateral leg; ROM, range of motion; MET, metabolic equivalent task; KOOS, Knee Injury and Osteoarthritis Outcome Score; NHANES, National Health And Nutrition Examination Survey; SF 36, Short Form-36 health survey; EQ-5D, EuroQol – 5 dimensions; EQ-VAS, EuroQol - Visual Analog Scale; RAPT, Risk Assessment and Prediction Tool.

**Figure 1:**
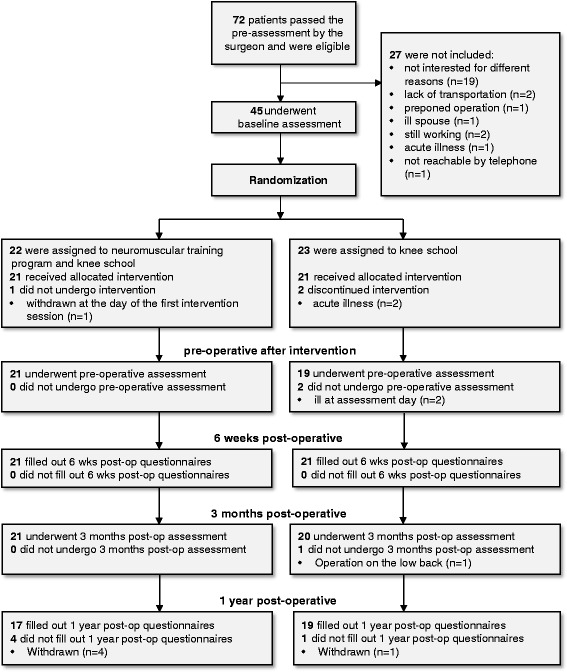
Flow diagram. Flow diagram of patients participating in this study.

Figure 1 shows the flow diagram of patients participating in this study.

### Description of exercise program performance

#### Progression

The mean training level at the first session was 1.17 (±0.4) and at the last session 1.40 (±0.6). In total, 28.6% of the patients increased their training level, 71.4% stayed at the start level and none deteriorated.

#### Self-reported pain before and after training sessions

The mean self-reported pain on a numerical rating scale from 0 to 10 was 2.79 (±1.7) immediately before training, 2.8 (±1.7) immediately after training and 3.1 (±1.7) one day after training. 63.6% of the patients in the exercise group reported increased pain twenty-four hours after training (3.4 (±1.6)). One patient had increased pain > 5 and missed the subsequent two sessions of training.

#### Adherence and joint-specific adverse events

The median number of attended sessions was 10 (IQR: 8, 14). 76.2% of the patients attended the pre-defined goal of 8 or more treatment sessions, while 23.8% attended less. The reasons for attending less than 8 sessions, were pre-scheduled surgery or in one patient withdrawal on the day of the first training session. In total, 231 (82%) of 282 sessions were attended and one patient missed 2 sessions due to increased pain, which was determined as a joint-specific adverse event.

### Additional objectively assessed physical activity

Besides the NEMEX-TJR, these patients reported an average activity performance of 17.9 hours per week (74% ADL activity, 25% endurance and strength, 1% sport).

### Treatment effects in the primary and secondary endpoints

#### After intervention but before surgery

We observed a small improvement for all primary and secondary endpoints in both exercise and control groups (see Table 2), which did not differ significantly between groups. Comparing the exercise to the control group the treatment effect for the Chair Stand Test was −1.5 seconds (95% CI: −5.3, 2.2), for KOOS ADL and KOOS pain the treatment effect was 1.3 points (−10.1, 12.8) and −2.3 (−12.4, 7.9) respectively.

**Table 2:** **Estimated treatment effects**

	**Baseline to 1 week pre-op**	**Baseline to 6 weeks post-op**	**Baseline to 3 months post-op**	**3 months to 12 months post-op**
**Primary endpoint**				
Chair Stand Test°	−1.5 (−5.3, 2.2)	Not assessed	2.0 (−1.8, 5.8)	Not assessed
**Secondary endpoints**				
KOOS ADL function°°	1.3 (−10.1, 12.8)	−2.0 (−13.3, 9.3)	−4.9 (−16.3, 6.5)	3.6 (−8.5, 15.7)
KOOS pain°°	−2.3 (−12.4, 7.9)	−6.4 (−16.5, 3.6)	−3.3 (−13.5, 6.8)	2.3 (−8.5, 13.0)
**Additional outcomes**				
**Lower limb function**				
KOOS symptoms°°	2.2 (−7.4, 11.8)	2.4 (−7.2, 11.9)	4.6 (−5.0, 14.2)	3.8 (−6.4, 14.0)
KOOS sport and recreation°°	−5.1 (−24.5, 14.4)	12.2 (−8.8, 33.2)	1.0 (-19.9, 21.8)	−4.1 (−25.6, 17.5)
KOOS quality of life°°	5.5 (−7.2, 18.1)	6.1 (−6.6, 18.7)	−5.9 (−18.5, 6.8)	5.9 (−7.5, 19.3)
Muscle strength (Newton)°		Not assessed		Not assessed
Knee extension op	8.8 (−40.0, 57.8)		−3.5 (−52.7, 45.6)	
Knee extension contra	14.2 (−29.8, 58.2)		35.4 (−8.9, 79.4)	
Knee flexion op	23.2 (−0.1, 46.5)		−12.7 (−36.2, 10.8)	
Knee flexion contra	2.0 (−20.8, 24.7)		−6.7 (−29.7, 16.2)	
Knee-bending/30s°	3.8 (−0.5, 8.0)	Not assessed	−3.3 (−7.4, 0.8)	Not assessed
ROM of the knee (degrees)°		Not assessed		Not assessed
Flexion op	1.9 (−4.4, 8.2)		−3.9 (−10.2, 2.4)	
Flexion contra	0.1 (−3.4, 3.7)		−1.6 (−5.2, 1.9)	
Extension op	0.8 (−2.4, 4.0)		1.4 (−1.8, 4.5)	
Extension contra	1.2 (−0.5, 2.8)		1.4 (−0.2, 3.0)	
20 m walk test°	−0.6 (−2.0, 0.9)	Not assessed	−0.5 (−2.0, 1.0)	Not assessed
Timed up and go°	0.2 (−1.5, 2.0)	Not assessed	1.6 (−0.1, 3.3)	Not assessed
**Physical activity**				
METs 7 day (kcal/h/kg)°	0.3 (−2.2, 2.7)	Not assessed	0.3 (−2.3, 2.9)	Not assessed
Steps (daily average)°	−687.2 (−2172, 798)	Not assessed	165.7 (−1288, 1620)	Not assessed
Adapted NHANES III METs°	10.0 (−12.6, 32.7)	1.6 (−20.9, 24.0)	0.4 (−22.3, 23.0)	2.6 (−21.3, 26.4)
**Health-related quality of life**				
SF 36 Physical functioning°°	7.1 (−5.3, 19.5)	0.5 (−11.8, 12.7)	−6.6 (−8.5, 17.5)	4.5 (−8.5, 17.5)
Role physical°°	−10.8 (−39.9, 18.3)	−5.2 (−34.2, 23.9)	−3.2 (−32.2, 25.9)	−1.1 (−31.7, 29.5)
Bodily pain°°	4.9 (−7.2, 17.0)	2.8 (−9.2, 14.9)	−3.4 (−15.5, 8.7)	4.9 (−7.8, 17.7)
General health°°	3.3 (−5.9, 12.4)	3.4 (−5.7, 12.5)	−2.8 (−12.0, 6.3)	2.5 (−7.2, 12.1)
Vitality°°	−2.3 (−13.9, 9.3)	−1.0 (−12.5, 10.5)	−8.3 (−20.0, 3.3)	3.3 (−8.9, 15.5.)
Social functioning°°	5.0 (−7.1, 17.2)	2.4 (−9.7, 14.4)	−1.6 (-13.7, 10.5)	−0.7 (−13.4, 12.1)
Role emotional°°	−10.8 (−34.3, 12.7)	−11.1 (−34.5, 12.3)	−10.2 (−34.0, 13.5)	−9.3 (−34.2, 15.7)
Mental health°°	2.6 (−6.6, 11.7)	−1.6 (−10.7, 7.5)	−3.0 (−12.2, 6.1)	0.4 (−9.2, 10.0)
EQ-5D Mobility°°°	0.1 (−0.3, 0.4)	0.0 (−0.3, 0.4)	−0.1 (−0.4, 0.2)	0.1 (−0.3, 0.4)
Self-care°°°	−0.0 (−0.2, 2.2)	0.1 (−0.2, 0.3)	0.1 (−0.1, 0.3)	0.0 (−0.2, 0.2)
Usual Activities°°°	−0.0 (−0.4, 0.4)	−0.1 (−0.5, 0.2)	0.0 (−0.4, 0.4)	−0.1 (−0.4, 0.3)
Pain/discomfort°°°	−0.1 (−0.4, 0.2)	0.1 (−0.3, 0.4)	−0.0 (−0.5, 0.3)	−0.1 (−0.4, 0.3)
Anxiety/Depression°°°	−0.1 (−0.3, 0.3)	0.0 (−0.2, 0.2)	−0.0 (−0.3, 0.3)	0.1 (−0.2, 0.3)
EQ-VAS°°	7.1 (−2.5, 16.7)	2.1 (−7.4, 11.6)	1.2 (−8.4, 10.8)	−0.1 (−10.2, 10.0)

Values are differences in mean changes between intervention and control group. Results from a linear mixed model and adjusted for age, gender and BMI.

° Continuous variable (time in seconds, Newton, degree, number, METs, steps).

°° Scores range from 0 to 100 with higher scores indicating fewer problems.

°°° Scores range from 1 to 3 with lower score indicating fewer problems.

#### 6 weeks after surgery

Comparing the exercise to the control group the treatment effect for KOOS ADL and KOOS pain was −2.0 points (−13.3, 9.3) and −6.4 (−16.5, 3.6) respectively.

#### Three months after surgery

Comparing the exercise to the control group the treatment effect in the Chair Stand Test was 2.0 seconds (−1.8, 5.8). For KOOS ADL and KOOS pain the treatment effect was −4.9 points (−16.3, 6.5) and −3.3 points (−13.5, 6.8) respectively.

#### 12 months after surgery compared to three months after surgery

Comparing the exercise to the control group the treatment effect for KOOS ADL and KOOS pain was 3.5 points (−8.5, 15.7) and 2.3 (−8.5, 13.0) respectively.

**Figure 2:**
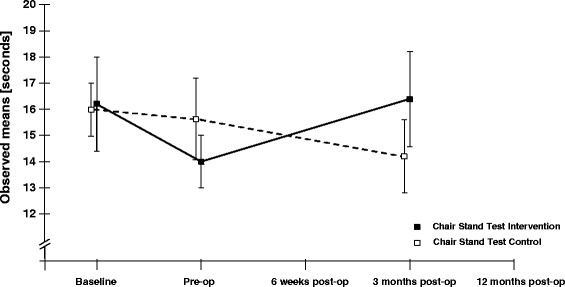
Primary endpoint results. Observed means +/− 1 Standard Error of the Chair Stand Test values for the two groups, at baseline, prior to surgery and 3 months after surgery. Baseline is treated as outcome, making no assumptions about group differences in the mean response at baseline, adjusted for age, gender and BMI.

**Figure 3:**
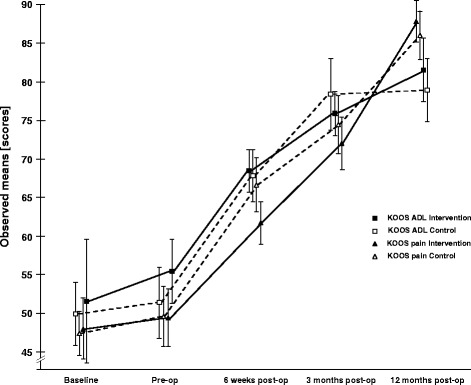
Secondary endpoint results. Observed means +/− 1 Standard Error of the KOOS ADL and KOOS pain scores for the two groups, at baseline, prior to surgery, 6 weeks, 3 months and 12 months after surgery. Baseline is treated as outcome, making no assumptions about group differences in the mean response at baseline, adjusted for age, gender and BMI.

All results are presented in Table 2, Figures 2 and 3.

### Treatment effects in the additional outcome measures

At all time-points, comparing the exercise to the control group we found no significant treatment effect in any additional outcome measures.

All results are presented in Table 2.

### Change within groups

In within groups we observed at 1 week preoperatively a significant improvement in the isometric muscle strength of the knee flexors of the operated leg in the exercise group (23.0 (−4.8, 50.8) and in the range of motion of the knee extension of the contralateral leg in the control group (−1.5 (−3.4, 0.5).

At 6 weeks postoperatively a significant improvement in all KOOS subscales beside symptoms was seen (KOOS ADL: 16.6 (0.7, 32.5) in the intervention group and 18.6 (2.9, 34.4) in the control group; KOOS pain: 13.2 (−1.0,27.3.) and 19.6. (5.6, 33.6) respectively; KOOS Sport/Rec: 38.3 (10.2, 66.3) and 26.1 (−4.7, 56.8) respectively; KOOS QOL: 25.5 (7.9, 43.1) and 19.4 (1.7, 37.2) respectively) as well as in some dimensions of the SF 36 (bodily pain: 15.8 (−1.0, 32.5) and 13.0 (−3.9, 29.8) respectively; general health: 11.2 (−1.4, 23.9) in the intervention group) and in EQ-5D mobility (−0.4 (−0.9, 0.1) and −0.4 (−0.9, 0.1) respectively).

At 3 months postoperatively we identified in both groups a significant improvement in all KOOS subscales beside symptoms in the control group (KOOS ADL (24.0 (8.1, 39.9) and 28.9 (12.9, 44.9) respectively; KOOS pain: 23.4, (9.3, 37.5) and 26.7 (12.5, 40.9) respectively; KOOS Sport/Rec: 35.7 (7.2, 64.2) and 34.7 (4.9, 64.6) respectively; KOOS QOL: 32.6 (15.0, 50.2) and 38.5 (20.8, 56.2) respectively; KOOS symptoms: 9.9 (−3.5, 23.3) in the intervention group), as well as in some dimensions of the SF-36 (physical functioning: 22.4 (5.3, 39.4) and 29.0 (11.5, 46.4) respectively; bodily pain: 24.5 (7.8, 41.2) and 27.9 (10.8, 45.0) respectively; general health: 11.8 (−0.8, 24.5) and 14.7 (1.8, 27.6) respectively; role physical: 31.2 (−9.9, 72.2) in the control group; vitality: 13.9 (−2.5, 30.3) in the control group; mental health: 9.5 (−3.5, 22.4) in the control group), the EQ-5D mobility (−0.5 (−1.0, −0.0) and −0.4 (−0.9, 0.1) respectively) and the EQ-VAS (13.9 (0.6, 27.1) in the intervention group.

Between 3 and 12 months postoperatively booth groups improved significantly in KOOS pain (15.2 (−0.0, 30.3) and 12.9 (−2.0, 27.7) respectively) and the intervention group improved also significantly in SF 36 bodily pain (13.9 (−4.1, 31.9).

All results are presented in Table 3.

**Table 3:** **Estimated within group changes**

	**Baseline to 1 week pre-op**		**Baseline to 6 weeks post-op**		**Baseline to 3 months post-op**		**3 months to 12 months post-op**	
**Primary endpoint**	**Intervention (n = 21)**	**Control (n = 19)**	**Intervention (n = 21)**	**Control (n = 21)**	**Intervention (n = 21)**	**Control (n = 20)**	**Intervention (17)**	**Control (19)**
Chair Stand Test°	−2.4 (−6.8, 2.1)	−0.8 (−5.4, 3.7)	not assessed	Not assessed	0.3 (−4.3, 4.8)	−1.7 (−6.3, 2.9)	not assessed	not assessed
**Secondary endpoints**								
KOOS ADL function°°	3.7 (−12.2, 19.5)	2.3 (−13.7, 18.3)	16.6 (0.7, 32.5)***	18.6 (2.9, 34.4)***	24.0 (8.1, 39.9)****	28.9 (12.9, 44.9)****	5.0 (−12.0, 22.1)	1.4 (−15.3, 18.2)
KOOS pain°°	0.8 (−13.3, 14.9)	3.0 (−11.2, 17.2)	13.2 (−1.0, 27.3)**	19.6 (5.6, 33.6)****	23.4 (9.3, 37.5)****	26.7 (12.5, 40.9)****	15.2 (−0.0, 30.3)**	12.9 (−2.0, 27.7)**
**Lower limb function**								
KOOS symptoms°°	1.7 ( −11.7, 15.1)	−0.5 (−14.0, 13.0)	7.3 (−6.1, 20.7)	5.0 (−8.3, 18.3)	9.9 (−3.5, 23.3)*	5.2 (−8.2, 18.7)	4.5 (−9.9, 18.9)	0.7 (−13.4, 14.8)
KOOS sport and recreation°°	1.0 (−26.4, 28.4)	6.1 (−20.9, 33.0)	38.3 (10.2, 66.3)****	26.1 (−4.7, 56.8)*	35.7. (7.2, 64.2)****	34.7 (4.9, 64.6)****	8.5 (−21.1, 38.1)	12.5 (−17.9, 43.0)
KOOS quality of life°°	3.8 (−13.8, 21.4)	−1.7 (−19.4, 16.0)	25.5 (7.9, 43.1)****	19.4 (1.7, 37.2)***	32.6 (15.0, 50.2)****	38.5 (20.8, 56.2)****	12.8 (−6.1, 31.7)	6.9 (−11.6, 25.5)
Muscle strength (Newton)°			not assessed	not assessed			not assessed	not assessed
Knee extension op	10.2 (−48.0, 68.4)	1.4 (−57.4, 60.3)			−47.1 (−106.2, 12.1)*	−43.5 (−102.3, 15.3)*		
Knee extension contra	14.7 (−22.3, 51.8)	0.6 (−37.0, 38.1)			−0.9 (−38.6, 36.9)	−36.3 (−73.8, 1.2)		
Knee flexion op	23.0 (−4.8, 50.8)*	−0.2 (−28.4, 27.9)			−20.9 (−49.2, 7.4)*	−8.2 (−36.3, 20.0)		
Knee flexion contra	9.1 (−18.0, 36.2)	7.1 (−20.4, 34.6)			−5.0 (−32.6, 22.7)	1.8 (−25.6, 29.3)		
Knee-bending/30s°	2.9 (−2.4, 8.2)	−0.9 (−6.0, 4.2)	not assessed	not assessed	0.4 (−4.6, 5.5)	3.7 (−1.2, 8.7)*	not assessed	not assessed
ROM of the knee (degrees)°			not assessed	not assessed			not assessed	not assessed
Flexion op	2.2. (−5.4, 9.7)	0.2 (−7.4, 7.8)			−2.6 (−10.1, 4.9)	1.3 (−6.3, 8.9)		
Flexion contra	1.2 (−3.0, 5.4)	1.0 (−3.2, 5.3)			0.9 (−3.3, 5.2)	2.6 (−1.7, 6.8)		
Extension op	0.6 (−3.2, 4.3)	−0.3 (−4.1, 3.6)			−2.7 (−6.4, 1.2)	−4.0 (−7.8, −0.2)**		
Extension contra	−0.4 (−2.3, 1.6)	−1.5 (−3.4, 0.5)*			−0.7 (−2.6, 1.2)	−2.1 (−4.0, −0.2)**		
20 m walk test°	−0.6 (−2.3, 1.6)	−0.0 (−1.8, 1.7)	not assessed	not assessed	−0.9 (−2.6, 0.9)	−0.4 (−2.2, 1.3)	not assessed	not assessed
Timed up and go°	−0.6 (−2.6, 1.5)	−0.8 (−2.9, 1.3)	not assessed	not assessed	0.4 (−1.7, 2.5)	−1.2 (−3.3, 0.9)	not assessed	not assessed
**Physical activity**								
METs 7 day (kcal/h/kg)	0.8 (−2.4, 3.4)	0.5 (−2.4, 3.4)	not assessed	not assessed	1.1 (−1.7, 3.9)	0.5 (−2.4, 3.5)	not assessed	not assessed
Steps (daily average)	−127.5 (−1932, 1677)	559.8 (−1201, 2321)	not assessed	not assessed	−130.3 (−1815, 1554)	−296.0 (−2103, 1512)	not assessed	not assessed
Adapted NHANES III METs	5.1 (−26.6, 36.9)	−4.9 (−36.4, 26.6)	−8.5 (−40.2, 23.3)	−10.1 (−41.1, 21.0)	6.5 (−25.3, 38.2)	6.2 (−25.4, 37.6)	12.2 (−21.5, 45.9)	9.6 (−23.4, 42.6)
**Health-related quality of life**								
SF 36 Physical functioning°°	2.4 (−14.8, 19.4)	−4.7 (−22.2, 12.7)	10.2 (−6.9, 27.3)	9.8 (−7.4, 27.0)	22.4 (5.3, 39.4)****	29.0 (11.5, 46.4)****	5.0 (−13.3, 23.4)	0.5 (−17.5, 18.5)
Role physical°°	−6.5 (−46.7, 33.6)	4.3 (−36.7, 45.3)	−4.1 (−44.3, 36.0)	1.0 (−40.0, 42.0)	28.0 (−12.1, 68.2)	31.2 (−9.9, 72.2)*	3.8 (−39.4, 47.0)	4.9 (−37.4, 47.2)
Bodily pain°°	1.6 (−15.1, 18.3)	−3.3 (−20.4, 13.8)	15.8 (−1.0, 32.5)**	13.0 (−3.9, 29.8)*	24.5 (7.8, 41.2)****	27.9 (10.8, 45.0)****	13.9 (−4.1, 31.9)*	9.0 (−8.6, 26.6)
General health°°	6.7 (−6.0, 19.3)	3.4 (−9.6, 16.3)	11.2 (−1.4, 23.9)**	7.8 (−4.9, 20.5)	11.8 (−0.8, 24.5)**	14.7 (1.8, 27.6)***	−4.2 (−17.7, 9.3)	−6.6 (−19.9, 6.6)
Vitality°°	0.1 (−16.0, 16.1)	2.4 (−14.0, 18.8)	3.0 (−13.1, 19.0)	3.9 (−12.2, 20.1)	5.6 (−10.4, 21.6)	13.9 (−2.5, 30.3)*	4.0 (−13.2, 21.2)	0.7 (−16.2, 17.6)
Social functioning°°	2.5 (−14.2, 19.3)	−2.5 (−19.6, 14.6)	4.3 (−12.4, 21.1)	2.0 (−14.9, 18.8)	6.7 (−10.1, 23.4)	8.3 (−8.8, 25.4)	−0.7 (−18.7, 17.4)	0.0 (−17.6, 17.7)
Role emotional°°	3.9 (−28.6, 36.5)	14.7 (−18.5, 48.0)	−2.4 (−35.0, 30.1)	8.7 (−24.1, 41.4)	5.5 (−27.0, 38.1)	15.7 (−18.1, 50.0)	−1.5 (−36.5, 33.4)	7.7 (−27.1, 42.5)
Mental health°°	1.3 (−11.3, 14.0)	−1.3 (−14.2, 11.6)	4.0 (−7.7, 17.5)	6.5 (−6.2, 19.2)	6.4 (−6.2, 19.1)	9.5 (−3.5, 22.4)*	−1.0 (−14.6, 12.5)	−1.4 (−14.7, 11.9)
EQ-5D Mobility°°°	−0.1 (−0.5, 0.4)	−0.1 (−0.6, 0.3)	−0.4 (−0.9, 0.1)**	−0.4 (−0.9, 0.05)**	−0.5 (−1.0, −0.0)***	−0.4 (−0.9, 0.1)*	−0.1 (−0.6, 0.4)	−0.2 (−0.7, 0.3)
Self-care°°°	0.1 (−0.2, 0.4)	0.1 (−0.2, 0.4)	0.1 (−0.2, 0.4)	0.0 (−0.3, 0.3)	−0.0 (−0.3, 0.3)	−0.1 (−0.4, 0.2)	−0.0 (−0.3, 0.3)	−0.0 (−0.3, 0.3)
Usual Activities°°°	0.0 (−0.4, 0.6)	0.0 (−0.4, 0.6)	0.0 (−0.4, 0.6)	0.0 (−0.3, 0.7)	−0.0 (−0.7, 0.3)	−0.0 (−0.7, 0.3)	−0.0 (−0.6, 0.4)	−0.0 (−0.6, 0.5)
Pain/discomfort°°°	0.0 (−0.4, 0.5)	0.1 (−0.4, 0.6)	−0.2 (−0.6, 0.3)	−0.3 (−0.7, 0.2)	−0.3 (−0.7, 0.2)	−0.3 (−0.7, 0.2)	−0.3 (−0.8, 0.2)	−0.2 (−0.7, 0.2)
Anxiety/Depression°°°	−0.0 (−0.3, 0.3)	0.0 (−0.3, 0.4)	−0.0 (−0.4, 0.3)	−0.0 (−0.4, 0.2)	−0.0 (−0.3, 0.3)	0.0 (−0.3, 0.4)	−0.0 (−0.4, 0.3)	−0.0 (−0.5, 0.2)
EQ-VAS°°	3.9 (−9.4, 17.1)	−3.2 (−16.8, 10.3)	8.1 (−5.1, 21.4)	6.0 (−7.3, 19.4)	13.9 (0.6, 27.1)***	12.6 (−0.9, 26.2)	−1.7 (−16.0, 12.5)	−1.6 (−15.6, 12.4)

Results from a linear mixed model and adjusted for age, gender and BMI. Values are the mean (95%CI). CI not containing zero indicate statistical significance.

****p <0.0001 ***p ≤ 0.001 **p ≤ 0.01 *p ≤ 0.05.

°Continuous variable (time in seconds, Newton, degree, number, METS, steps).

°°Scores range from 0 to 100 with higher scores indicating fewer problems.

°°°Scores range from 1 to 3 with lower score indicating fewer problems.

## Discussion

This randomized, assessor-blinded, controlled trial evaluated the effect of a preoperative neuromuscular training (NEMEX-TJR) in addition to patient education compared to patient education alone of patients undergoing TKR on functional outcomes at 3 months postoperatively. We could not confirm our hypothesis that patients undergoing the NEMEX-TJR have improve in functional outcome at 3 months after surgery. We observed no improvement in the primary endpoint and a significant improvement in the secondary endpoints in both exercise and control groups, which did not differ significantly between groups. Noticeably, after the intervention but before surgery we observed a small improvement for all primary and secondary endpoints in both exercise and control groups, which also did not differ significantly between groups.

The programme was well tolerated based on our adverse event assessment and probably offered some benefit to the patients.

After the intervention but before surgery we found a small and non-significant benefit in favour of the exercise group. In support, a Danish trial using the same exercise intervention with a higher average number of training sessions (13 compared to 10) found a moderate and significant effect in favour of the exercise group. However this trial included both hip and knee patients and a sub group analysis revealed that the improvement in the hip patients was driving the overall effect [47]. Our results are in line with a meta-analysis of four trials with 240 participants [48], which provided moderate quality of evidence that exercise interventions compared with standard care were effective in reducing pain from knee osteoarthritis prior to knee replacement. In addition, our trial showed improvement in performed function which is in disagreement with the meta-analysis.

At 6 weeks postoperatively we found no between-group differences in KOOS ADL and pain which is in contrast with the Danish trial, where the authors found significantly greater improvements in ADL and pain from exercise [49]. Other studies showed comparable within group results for pain and self-reported function in their exercise and control groups from preoperative time point through 6–8 week postoperative follow up [50,51].

At 3 months postoperatively (primary endpoint), no additional benefits were seen from the preoperative neuromuscular training. This is consistent with the Danish trial where the faster post-operative improvement in ADL and pain from exercise was blunted after surgery at 3 months follow-up [49]. Two other RCTs, evaluating the effect of preoperative training on post-operative outcome, also reported only within group changes without between group differences [52,53]. Interestingly the positive trajectory of self-reported KOOS ADL scores in both groups does not reflect the non-improvement or the worsening in the objective CST. Several studies confirm these results, noting improvement in self-reported measures but not in performance tests [54-56].

We formulated no hypothesis for 12 months postoperative because, based on literature, we did not expect a significant difference between-groups mean changes, since, over the long term, the effects of exercise and control group on functional performance seem to converge [57]. Yet, we found significant within-group improvements in both groups, but no between-group difference in KOOS pain from 3 months to 12 months postoperatively.

The performance level of our study population with respect to the Chair Stand Test was similar to other studies with the same patient group at baseline (16.3 ± 8.3 in our study compared to 14.3 ± 6.8 and 13.5 ± 5.9) [21,49]. It was therefore unexpected, that Chair Stand Test performance did not improve significantly in the exercise group at 3 months compared to baseline. An explanation might be that patients used the contralateral leg as a compensation to complete the function task [58]. Also, the KOOS showed similar results in all five subscales at baseline compared with other studies of the same patient population [21,49,59]. 3 months postoperatively, we identified statistically significant differences between groups in KOOS ADL, pain and QOL of 3 – 6 points in favour of the exercise group. The question is whether this difference at group level calls for the implementation of preoperative exercise in clinical practice. It is increasingly recognized that minimal important change (MIC) is dependent on context factors, such as patient characteristics, type of intervention, time to follow-up, dimension evaluated, method applied to calculate the MIC, and cut-off chosen for the anchor question [60]. As a consequence, “there is no universal MIC, despite the appeal of the notion” [60]. Additionally it should be kept in mind that the MIC can be calculated both at an individual level for use in clinical decision making, and at a group level for use in research. In research, hypothesis testing and statistical analysis is used to determine the result, and in this study the average minimal important difference (MID) between groups ranged from 3–6 points. These between-group differences of 3–6 points are greater than the smallest detectable differences reported on a group level (1.3-2.4) in the only available study giving these data relating to the KOOS [61], indicating that the current study is most probably powered to detect between group differences significantly different from zero. It should be kept in mind, however, that there is a lack of studies stating the measurement error in terms of the smallest detectable difference for groups in older patients or those having a TKR.

In our study, four senior orthopaedic surgeons performed all operations, but surgical procedures were not optimally standardized, which might be a possible cause of bias. It was also not possible to standardize the rehabilitation procedure in an optimal form, meaning that some of the patients were discharged to an inpatient rehabilitation facility and others were discharged home and received treatment in an outpatient physiotherapy practice. That could also be a source of bias.

Our study has several strengths. First, our RCT was conducted according to the CONSORT statement [62,63] with a rigorous study design, a clinically feasible intervention and good adherence to the programme. Inclusion criteria were kept broad to reflect daily clinical practice as far as possible. Second, the blinding of the assessor was ensured through restriction of access to obtained data and patient discretion. Third, we assessed outcomes over the entire time period from baseline to 1 week preoperative and 6 weeks, 3 months and 12 months postoperative respectively. Further, the conducted intervention was safe. Moreover, to control of attention bias, we offered a knee school for all participants.

There are also some limitations to our trial, including the relatively small recruited sample size of 45 patients instead of the planned 80 patients. Because of a change in the reimbursement system in the acute care hospitals, a reduction in preoperative waiting-time occurred during the recruitment phase, thus, our trial is underpowered due to logistical recruitment problems that we had to adapt to. Additionally, our assumptions made in November 2008 for calculating the sample size seem, in retrospect, and in the light of several recently published studies, too optimistic [21,47]. The presented *p*-values und confidence intervals in this study will help the reader to interpret our data realistically. Further, we assumed that functional status of all patients was reduced and a chance of improvement plausible. However, only 16 out of 21 patients started at the lowest exercise level when starting with the NEMEX-TJR. This may have been avoided by excluding patients with higher functional levels. On the other hand the preoperative KOOS scores of our participants are consistent with the literature of patients undergoing TRK [21,47,49]. Also, due to slow recruitment, most training did not take place in groups and the expected positive effect of group training, in terms of more effective learning compared with individual practice sessions, was therefore diminished [64]. Moreover, the median of completed sessions in our study was 10, which is at the lower limit in terms of adequate dosage [15] and only 19% of patients progressed to a more difficult training level, although self-reported pain seemed not to be a limiting factor. In comparison to previous studies in the Scandinavian countries [21,49], very few patients progressed to more difficult training levels. This may reflect a cultural difference among patients or therapists in the role of exercise as osteoarthritis treatment. Delivering a sham exercise instead of a knee school educational programme would have been optimal, but was not realistic due to the difficulty in designing a credible placebo intervention.

## Conclusions

Viewed over the entire period from baseline to 12 months postoperatively, a median (IQR) of 10 (8, 14) exercise sessions (NEMEX-TJR) before surgery showed only a small and non-significant improvement in all functional assessments compared to patient education alone. Also, this benefit was not maintained after surgery.

Out trial doesn’t give a conclusive answer to whether additional preoperative exercise on postoperative functional outcome is beneficial. However, we acknowledge the small size of our trial as a limitation that may have prevented us to document small and sustained benefits.

## Additional file

Additional file 1:
**Details of the model.**

## References

[1] Harris WH, Sledge CB (1990). Total hip and total knee replacement (2). N Engl J Med.

[2] Guccione AA, Felson DT, Anderson JJ, Anthony JM, Zhang Y, Wilson PW (1994). The effects of specific medical conditions on the functional limitations of elders in the Framingham Study. Am J Public Health.

[3] Felson DT, Lawrence RC, Dieppe PA, Hirsch R, Helmick CG, Jordan JM (2000). Osteoarthritis: new insights. Part 1: the disease and its risk factors. Ann Intern Med.

[4] Seed SM, Dunican KC, Lynch AM (2009). Osteoarthritis: a review of treatment options. Geriatrics.

[5] Kurtz SM, Ong KL, Lau E, Widmer M, Maravic M, Gomez-Barrena E (2011). International survey of primary and revision total knee replacement. Int Orthop.

[6] Meneghini RM, Russo GS, Lieberman JR (2014). Modern perceptions and expectations regarding total knee arthroplasty. J Knee Surg.

[7] Hamilton DF, Clement ND, Burnett R, Patton JT, Moran M, Howie CR (2013). Do modern total knee replacements offer better value for money? A health economic analysis. Int Orthop.

[8] Beswick AD, Wylde V, Gooberman-Hill R, Blom A, Dieppe P (2012). What proportion of patients report long-term pain after total hip or knee replacement for osteoarthritis? A systematic review of prospective studies in unselected patients. BMJ Open.

[9] Alzahrani K, Gandhi R, Debeer J, Petruccelli D, Mahomed N (2011). Prevalence of clinically significant improvement following total knee replacement. J Rheumatol.

[10] Judge A, Welton NJ, Sandhu J, Ben-Shlomo Y (2010). Equity in access to total joint replacement of the hip and knee in England: cross sectional study. BMJ.

[11] Singh JA, O’Byrne MM, Harmsen WS, Lewallen DG (2010). Predictors of moderate-severe functional limitation 2 and 5 years after revision total knee arthroplasty. J Arthroplasty.

[12] Scott CE, Howie CR, MacDonald D, Biant LC (2010). Predicting dissatisfaction following total knee replacement: a prospective study of 1217 patients. J Bone Joint Surg.

[13] Hoogeboom TJ, van den Ende CH, van der Sluis G, Elings J, Dronkers JJ, Aiken AB (2009). The impact of waiting for total joint replacement on pain and functional status: a systematic review. Osteoarthritis Cartilage.

[14] Mancuso CA, Graziano S, Briskie LM, Peterson MG, Pellicci PM, Salvati EA (2008). Randomized trials to modify patients’ preoperative expectations of hip and knee arthroplasties. Clin Orthop Relat Res.

[15] Hoogeboom TJ, Oosting E, Vriezekolk JE, Veenhof C, Siemonsma PC, de Bie RA (2012). Therapeutic validity and effectiveness of preoperative exercise on functional recovery after joint replacement: a systematic review and meta-analysis. PLoS One.

[16] Beckwee D, Vaes P, Cnudde M, Swinnen E, Bautmans I (2013). Osteoarthritis of the knee: why does exercise work? A qualitative study of the literature. Ageing Res Rev.

[17] Bennell KL, Wrigley TV, Hunt MA, Lim BW, Hinman RS (2013). Update on the role of muscle in the genesis and management of knee osteoarthritis. Rheum Dis Clin North Am.

[18] Ageberg E, Link A, Roos EM (2010). Feasibility of neuromuscular training in patients with severe hip or knee OA: the individualized goal-based NEMEX-TJR training program. BMC Musculoskelet Disord.

[19] Johansson K, Nuutila L, Virtanen H, Katajisto J, Salantera S (2005). Preoperative education for orthopaedic patients: systematic review. J Adv Nurs.

[20] Nunez M, Nunez E, Segur JM, Macule F, Quinto L, Hernandez MV (2006). The effect of an educational program to improve health-related quality of life in patients with osteoarthritis on waiting list for total knee replacement: a randomized study. Osteoarthritis Cartilage.

[21] Ageberg E, Nilsdotter A, Kosek E, Roos EM (2013). Effects of neuromuscular training (NEMEX-TJR) on patient-reported outcomes and physical function in severe primary hip or knee osteoarthritis: a controlled before-and-after study. BMC Musculoskelet Disord.

[22] Huber EO, de Bie RA, Roos EM, Bischoff-Ferrari HA (2013). Effect of pre-operative neuromuscular training on functional outcome after total knee replacement: a randomized-controlled trial. BMC Musculoskelet Disord.

[23] Bohannon RW (1998). Alternatives for measuring knee extension strength of the elderly at home. Clin Rehabil.

[24] Bohannon RW (1995). Sit-to-stand test for measuring performance of lower extremity muscles. Percept Mot Skills.

[25] Lord SR, Murray SM, Chapman K, Munro B, Tiedemann A (2002). Sit-to-stand performance depends on sensation, speed, balance, and psychological status in addition to strength in older people. J Gerontol A Biol Sci Med Sci.

[26] Villadsen A, Roos EM, Overgaard S, Holsgaard-Larsen A (2012). Agreement and reliability of functional performance and muscle power in patients with advanced osteoarthritis of the hip or knee. Am J Phys Med Rehabil.

[27] Guralnik JM, Ferrucci L, Simonsick EM, Salive ME, Wallace RB (1995). Lower-extremity function in persons over the age of 70 years as a predictor of subsequent disability. N Engl J Med.

[28] Collins NJ, Misra D, Felson DT, Crossley KM, Roos EM (2011). Measures of knee function: International Knee Documentation Committee (IKDC) Subjective Knee Evaluation Form, Knee Injury and Osteoarthritis Outcome Score (KOOS), Knee Injury and Osteoarthritis Outcome Score Physical Function Short Form (KOOS-PS), Knee Outcome Survey Activities of Daily Living Scale (KOS-ADL), Lysholm Knee Scoring Scale, Oxford Knee Score (OKS), Western Ontario and McMaster Universities Osteoarthritis Index (WOMAC), Activity Rating Scale (ARS), and Tegner Activity Score (TAS). Arthritis Care Res (Hoboken).

[29] Roos EM (2003). Effectiveness and practice variation of rehabilitation after joint replacement. Curr Opin Rheumatol.

[30] Kessler S, Lang S, Puhl W, Stove J (2003). The Knee Injury and Osteoarthritis Outcome Score–a multifunctional questionnaire to measure outcome in knee arthroplasty. Z Orthop Ihre Grenzgeb.

[31] Stoll T, Huber E, Seifert B, Michel BA, Stucki G (2000). Maximal isometric muscle strength: normative values and gender-specific relation to age. Clin Rheumatol.

[32] Huber E, Stoll T, Ehrat B, Hofer HO, Seifert N, Stucki G (1997). Zuverlässigkeit und Normperzentilen einer neuen isometrischen Muskelkraftmessmethode. Physiotherapie SPV.

[33] Hortobagyi T, Garry J, Holbert D, Devita P (2004). Aberrations in the control of quadriceps muscle force in patients with knee osteoarthritis. Arthritis Rheum.

[34] Roos EM, Bremander AB, Englund M, Lohmander LS (2008). Change in self-reported outcomes and objective physical function over 7 years in middle-aged subjects with or at high risk of knee osteoarthritis. Ann Rheum Dis.

[35] Lenssen AF, van Dam EM, Crijns YH, Verhey M, Geesink RJ, van den Brandt PA (2007). Reproducibility of goniometric measurement of the knee in the in-hospital phase following total knee arthroplasty. BMC Musculoskelet Disord.

[36] Faulkner KA, Redfern MS, Rosano C, Landsittel DP, Studenski SA, Cauley JA (2006). Reciprocal influence of concurrent walking and cognitive testing on performance in older adults. Gait Posture.

[37] Podsiadlo D, Richardson S (1991). The timed “Up & Go”: a test of basic functional mobility for frail elderly persons. J Am Geriatr Soc.

[38] Pitta F, Troosters T, Probst VS, Spruit MA, Decramer M, Gosselink R (2006). Quantifying physical activity in daily life with questionnaires and motion sensors in COPD. Eur Respir J.

[39] Holsgaard-Larsen A, Roos EM (2012). Objectively measured physical activity in patients with end stage knee or hip osteoarthritis. Eur J Phys Rehabil Med.

[40] Nelson KM, Reiber G, Boyko EJ (2002). Diet and exercise among adults with type 2 diabetes: findings from the third national health and nutrition examination survey (NHANES III). Diabetes Care.

[41] Ainsworth BE, Haskell WL, Herrmann SD, Meckes N, Bassett DR, Tudor-Locke C (2011). 2011 Compendium of Physical Activities: a second update of codes and MET values. Med Sci Sports Exerc.

[42] Ware JE (1993). Measuring patients’ views: the optimum outcome measure. BMJ.

[43] Ware JE, Sherbourne CD (1992). The MOS 36-item short-form health survey (SF-36). I. Conceptual framework and item selection. Med Care.

[44] Rabin R, de Charro F (2001). EQ-5D: a measure of health status from the EuroQol Group. Ann Med.

[45] R Core Team. R: A language and environment for statistical computing. [http://www.R-project.org/.]

[46] lme4: Linear mixed-effects models using Eigen and S4. R package version 1.1-6. [http://CRAN.R-project.org/package=lme4].

[47] Villadsen A, Overgaard S, Holsgaard-Larsen A, Christensen R, Roos EM (2014). Immediate efficacy of neuromuscular exercise in patients with severe osteoarthritis of the hip or knee: a secondary analysis from a randomized controlled trial. J Rheumatol.

[48] Wallis JA, Taylor NF (2011). Pre-operative interventions (non-surgical and non-pharmacological) for patients with hip or knee osteoarthritis awaiting joint replacement surgery–a systematic review and meta-analysis. Osteoarthritis Cartilage.

[49] Villadsen A, Overgaard S, Holsgaard-Larsen A, Christensen R, Roos EM (2014). Postoperative effects of neuromuscular exercise prior to hip or knee arthroplasty: a randomised controlled trial. Ann Rheum Dis.

[50] Rooks DS, Huang J, Bierbaum BE, Bolus SA, Rubano J, Connolly CE (2006). Effect of preoperative exercise on measures of functional status in men and women undergoing total hip and knee arthroplasty. Arthritis Rheum.

[51] McKay C, Prapavessis H, Doherty T (2012). The effect of a prehabilitation exercise program on quadriceps strength for patients undergoing total knee arthroplasty: a randomized controlled pilot study. PM R.

[52] Gstoettner M, Raschner C, Dirnberger E, Leimser H, Krismer M (2011). Preoperative proprioceptive training in patients with total knee arthroplasty. Knee.

[53] Topp R, Swank AM, Quesada PM, Nyland J, Malkani A (2009). The effect of prehabilitation exercise on strength and functioning after total knee arthroplasty. PM R.

[54] Parent E, Moffet H (2002). Comparative responsiveness of locomotor tests and questionnaires used to follow early recovery after total knee arthroplasty. Arch Phys Med Rehabil.

[55] Maly MR, Costigan PA, Olney SJ (2006). Determinants of self-report outcome measures in people with knee osteoarthritis. Arch Phys Med Rehabil.

[56] Stratford PW, Kennedy DM (2006). Performance measures were necessary to obtain a complete picture of osteoarthritic patients. J Clin Epidemiol.

[57] Farquhar SJ, Reisman DS, Snyder-Mackler L (2008). Persistence of altered movement patterns during a sit-to-stand task 1 year following unilateral total knee arthroplasty. Phys Ther.

[58] Mizner RL, Snyder-Mackler L (2005). Altered loading during walking and sit-to-stand is affected by quadriceps weakness after total knee arthroplasty. J Orthop Res.

[59] Holm B, Bandholm T, Lunn TH, Husted H, Aalund PK, Hansen TB (2014). Role of preoperative pain, muscle function, and activity level in discharge readiness after fast-track hip and knee arthroplasty. Acta Orthop.

[60] King MT (2011). A point of minimal important difference (MID): a critique of terminology and methods. Expert Rev Pharmacoecon Outcomes Res.

[61] Paradowski PT, Witonski D, Keska R, Roos EM (2013). Cross-cultural translation and measurement properties of the Polish version of the Knee injury and Osteoarthritis Outcome Score (KOOS) following anterior cruciate ligament reconstruction. Health Qual Life Outcomes.

[62] Moher D, Hopewell S, Schulz KF, Montori V, Gotzsche PC, Devereaux PJ (2010). CONSORT 2010 Explanation and Elaboration: Updated guidelines for reporting parallel group randomised trials. J Clin Epidemiol.

[63] Altman DG, Schulz KF, Moher D, Egger M, Davidoff F, Elbourne D (2001). The revised CONSORT statement for reporting randomized trials: explanation and elaboration. Ann Intern Med.

[64] McNevin NH, Wulf G, Carlson C (2000). Effects of attentional focus, self-control, and dyad training on motor learning: implications for physical rehabilitation. Phys Ther.

