# Supplementary material for: Effect of preoperative neuromuscular training (NEMEX-TJR) on functional outcome after total knee replacement: an assessor-blinded randomized controlled trial
Source: BMC Musculoskelet Disord. 2015 Apr 25;16:101. doi: 10.1186/s12891-015-0556-8 (PMC4414282; doi:10.1186/s12891-015-0556-8)
Supplement: Additional file 1: — Details of the model. [file 12891_2015_556_MOESM1_ESM.pdf]

Additional file 1: Details of the model

The model for observation Y_{ijk} (jth measure of subject k in group i) was $Y_{ijk} = \mu + \alpha_i + \beta_j + (\alpha\beta)_{ij} + U_{ik}$ + covariates + ϵ_{ijk}, $i = 1, 2$; $j = 1, ..., 5(3)$; $k = 1, ..., n_i$, with α_i as the ith group effect (time averaged group difference), β_j as the jth time effect (group averaged time differences), $(\alpha\beta)_{ij}$ as the ijth group-time interaction (difference between the within-group changes, equivalent to the treatment effect), U_{ik} as the random intercept of subject k in group i and ϵ_{ijk} as measurement error. For our LLM, we assumed that the U_{ik} and the ϵ_{ijk} are each independent and normally distributed with between-subject variance v^2 and within-subject variance τ^2, respectively, leading to a uniform within-subject correlation between two different measurements on the same subject.
